# Yanagi: Fast and interpretable segment-based alternative splicing and gene expression analysis

**DOI:** 10.1186/s12859-019-2947-6

**Published:** 2019-08-13

**Authors:** Mohamed K Gunady, Stephen M Mount, Héctor Corrada Bravo

**Affiliations:** 1 Department of Computer Science, University of Maryland, College Park, Maryland USA; 2 Center for Bioinformatics and Computational Biology, University of Maryland, College Park, Maryland USA; 3 Department of Cell Biology and Molecular Genetics, University of Maryland, College Park, Maryland USA

**Keywords:** Transcriptome quantification, Differential gene expression, Alternative splicing, RNA-seq, Pseudo-alignment

## Abstract

**Background:**

Ultra-fast pseudo-alignment approaches are the tool of choice in transcript-level RNA sequencing (RNA-seq) analyses. Unfortunately, these methods couple the tasks of pseudo-alignment and transcript quantification. This coupling precludes the direct usage of pseudo-alignment to other expression analyses, including alternative splicing or differential gene expression analysis, without including a non-essential transcript quantification step.

**Results:**

In this paper, we introduce a transcriptome segmentation approach to decouple these two tasks. We propose an efficient algorithm to generate maximal disjoint segments given a transcriptome reference library on which ultra-fast pseudo-alignment can be used to produce per-sample segment counts. We show how to apply these maximally unambiguous count statistics in two specific expression analyses – alternative splicing and gene differential expression – without the need of a transcript quantification step. Our experiments based on simulated and experimental data showed that the use of segment counts, like other methods that rely on local coverage statistics, provides an advantage over approaches that rely on transcript quantification in detecting and correctly estimating local splicing in the case of incomplete transcript annotations.

**Conclusions:**

The transcriptome segmentation approach implemented in *Yanagi* exploits the computational and space efficiency of pseudo-alignment approaches. It significantly expands their applicability and interpretability in a variety of RNA-seq analyses by providing the means to model and capture local coverage variation in these analyses.

**Electronic supplementary material:**

The online version of this article (10.1186/s12859-019-2947-6) contains supplementary material, which is available to authorized users.

## Background

Messenger RNA transcript abundance estimation from RNA-seq data is a crucial task in high-throughput studies that seek to describe the effect of genetic or environmental changes on gene expression. Transcript-level analysis and abundance estimation can play a central role in both fine-grained analysis of local splicing events and global analysis of changes in gene expression.

Over the years, various approaches have addressed the joint problems of (gene level) transcript expression quantification and differential alternative RNA processing. Much effort in the area has been dedicated to the problem of efficient alignment, or pseudo-alignment, of reads to a genome or a transcriptome, since this is typically a significant computational bottleneck in the analytical process starting from RNA-seq reads to produce gene-level expression or differentially expressed transcripts. Among these approaches are alignment techniques such as Bowtie [1], Tophat [2, 3], and Cufflinks [4], and newer techniques such as sailfish [5], RapMap [6], Kallisto [7] and Salmon [8], which provide efficient strategies through k-mer counting that are much faster, but maintain comparable, or superior, accuracy.

These methods simplified the expected outcome of the alignment step to only find sufficient read-alignment information required by the transcript quantification step. Given a transcriptome reference, an index of k-mers is created and used to find a mapping between reads and the list of compatible transcripts based on each approach’s definition of compatibility. The next step, quantification, would be to resolve the ambiguity in reads that were mapped to multiple transcripts. Many reads will multi-map to shared regions produced by alternative splicing even if free from error. The ambiguity in mapping reads is resolved using probabilistic models, such as the EM algorithm, to produce the abundance estimate of each transcript [9]. It is at this step that transcript-level abundance estimation faces substantial challenges that inherently affect the underlying analysis.

**Fig. 1:**
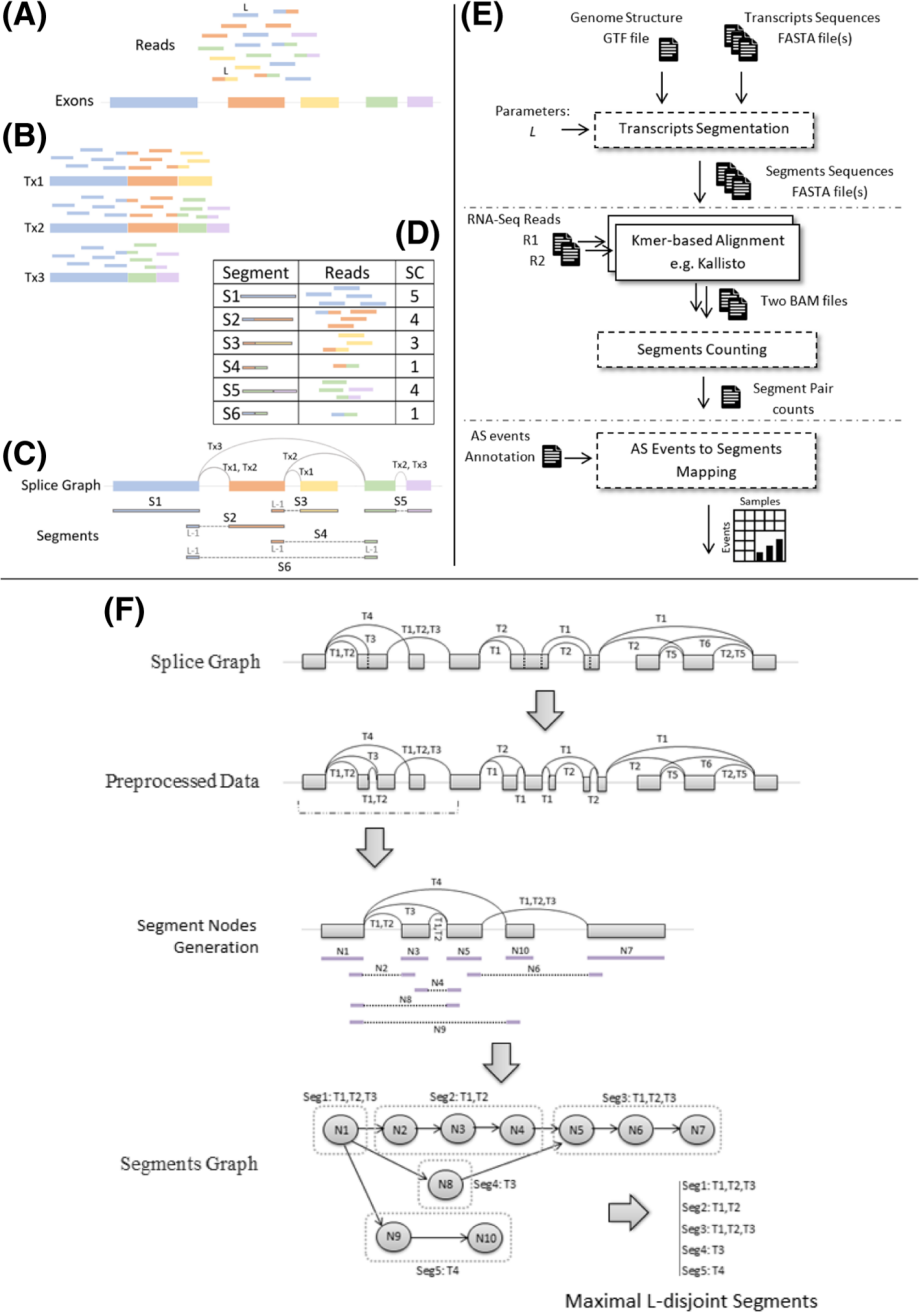
An overview of transcriptome segmentation and Yanagi-based workflow. (**a**) Shows the example set of exons and its corresponding sequenced reads. (**b**) shows the result of alignment over the annotated three isoforms spliced from the exons. (**c**) shows the splice graph representation of the three isoforms along with the generated segments from yanagi. (**d**) shows the alignment outcome when using the segments, and its segment counts (SCs). (**e**) Yanagi-based workflow: segments are used to align a paired-end sample then use the segments counts for downstream alternative splicing analysis. Dotted blocks are components of Yanagi. (**f**) Yanagi’s three steps for generating segments starting from the splice graph for an example of a complex splicing event. Assuming no short exons for simplicity. Step two and three are cropped to include only the beginning portion of the graph for brevity

Sequence repeats and paralogous genes can create ambiguity in the placement of reads. But more importantly, the fact that alternatively spliced isoforms share substantial portions of their coding regions, greatly increases the proportion of reads coming from these shared regions and, consequently, reads are frequently multi-mapped when aligning to annotated transcripts (Fig. 1**a**-**b**). In fact, local splicing variations can be joined combinatorially to create a very large number of possible transcripts from many genes. An extreme case is the Drosophila gene Dscam, which can produce over 38,000 transcripts by joining less than 50 exons [10]. Long-read sequencing indicates that a large number of possible splicing combinations is typical even in the presence of correlations between distant splicing choices [11].

Standard annotations, which enumerate only a minimal subset of transcripts from a gene (e.g. [12]), are thus inadequate descriptions. Furthermore, short read sequencing, which is likely to remain the norm for some time, does not provide information of long-range correlations between splicing events.

In this paper, we propose a novel strategy based on the construction and use of a transcriptome sequence segment library that can be used, without loss of information, in place of the whole transcriptome sequence library in the read-alignment-quantification steps. The segment library can fully describe individual events (primarily local splicing variation, but also editing sites or sequence variants) independently, leaving the estimation of transcript abundances through quantification as a separate problem. Here we introduce and formalize the idea of transcriptome segmentation, and propose and analyze an algorithm for transcriptome segmentation, implemented with a tool called Yanagi. To show how the segment library and segment counts can be used in downstream analysis, we show results from gene-level and alternative splicing differential analyses.

We propose the use of pseudo-alignment to calculate segment-level counts as a computationally efficient data reduction technique for RNA-seq data that yields sufficient intepretable information for a variety of downstream gene expression analysis.

## Results

### Yanagi’s Workflow for RNA-seq analysis

Figure 1**e** gives an overview of a Yanagi-based workflow which consists of three steps. The first step is the transcriptome segmentation, in which the segment library is generated. Given the transcriptome annotation and the genome sequences, Yanagi generates the segments in FASTA file format. This step of library preparation – done once and independently from the RNA-seq samples – requires a parameter value *L* which specifies the *m*aximum overlap length of the generated segments. The second step is pseudo-alignment. Using any k-mer based aligner (*e.g.* Kallisto or RapMap), the aligner uses the segments library for library indexing and alignment. The outcome of this step is read counts per segment (in case of single-end reads) or segment-pair counts (in case of paired-end reads). These segment counts (SCs) are the statistics that Yanagi provides for downstream analysis. The third step depends on the specific target analysis. On later subsections, we describe two use cases where using segment counts shows to be computationally efficient and statistically beneficial.

### Analysis of Generated Segments

For practical understanding of the generated segments, we used Yanagi to build segment libraries for the Drosophila melanogaster and Homo sapiens genome assemblies and annotations. These organisms show different genome characteristics, *e.g.* the fruit fly genome has longer exons than the human genome, while the number of annotated transcripts per gene is much higher for the human genome. A summary of the properties of each genome is found in [13].

#### Sequence lengths of generated segments

Segments generated by Yanagi’s approach are *L*-disjoint segments (See “Segments Properties” section). Since *L* is the only parameter required by the segmentation algorithm, we tried different values of *L* to understand the impact of that choice on the generated segments library. As mentioned in “Segments Properties” section, a proper choice of *L* is based on the expected read length of the sequencing experiment. For this analysis we chose the set *L*=(40,100,1000,10000) as a wide span of possible values of *L*.

Additional file 1: Figure S1 shows the histogram of the lengths of the generated segments compared to the histogram of the transcripts lengths, for each value of *L*, for both fruit fly (left) and human (right) genomes. The figure shows the expected behavior when increasing the value of *L*; using small values of *L* tends to shred the transcriptome more (higher frequencies for small sequence lengths), especially with genomes of complex splicing structure like the human genome. With high values of *L*, such as *L*=10,000, segments representing full transcripts are generated since the specificed minimum segment length tends to be longer than the length of most transcripts. It is important to note that the parameter *L* does not define the segments length since a segment length is mainly determined based on the neighboring branches in the splicing graph (See “Segments Properties” section), but rather *L* defines the maximum overlap allowed between segments, hence in a sense controls the minimum segment length (excluding trivial cases where the transcript itself is shorter than L).

#### Number of generated segments per gene

Additional file 1: Figure S2 shows how the number of generated segments in a gene is compared to the number of the transcripts in that gene, for each value of *L*, for both fruit fly (left) and human (right) genomes. A similar behavior is observed while increasing the value *L*, as with the segment length distribution. The fitted line included in each scatter plot provides indication of how the number of target sequences grows compared to the original transcriptome. For example, when using *L*=100 (a common read length with Illumina sequencing), the number of target sequences per gene, which will be the target of the subsequent pseudo-alignment steps, almost doubles. It is clear from both figures the effect of the third step in the segmentation stage. It is important not to shred the transcriptome so much that the target sequences become very short leading to complications in the pseudo-alignment and quantification steps, and not to increase the number of target sequences increasing the processing complexity of these steps.

#### Library Size of the generated segments

As a summary, Table 1 shows the library size when using segments compared to the reference transcriptome in terms of the total number of sequences, sequence bases, and file sizes. The total number of sequence bases clearly shows the advantage of using segments to reduce repeated sequences appearing in the library that corresponds to genomic regions shared among multiple isoforms. For instance, using *L*=100 achieves 54% and 35% compression rates in terms of sequence lengths for fruit-fly and human genomes, respectively. The higher the value of *L* is, the more overlap is allowed between segments, hence providing less the compression rate. Moreover, that necessarily hints to the expected behavior of the alignment step in terms of the frequency of multi-mappings.

**Table 1:** Library size summary when using segments compared to the reference transcriptome in terms of the total number of sequences, number of sequence bases, and total FASTA file sizes

	Transcriptome		Segments			
			*L*=40	*L*=100	*L*=1000	*L*=10000
BDGP6						
Number of bases (Gb)	90		39	41	71	90
Number of Sequences	34,681		54,680	53,694	48,741	34,625
FASTA File Size (MB)	89		44	47	76	92
GRCh38						
Number of bases (Gb)	278		147	181	308	281
Number of Sequences	182,435		544,991	541,361	264,083	183,165
FASTA File Size (MB)	276		206	239	338	302

With *L*=100, using segments achieves 54% and 35% compression rates over the transcriptome in terms of number of bases for fruit fly and human genomes, respectively.

#### Impact of using segments on Multi-mapped Reads

**Fig. 2:**
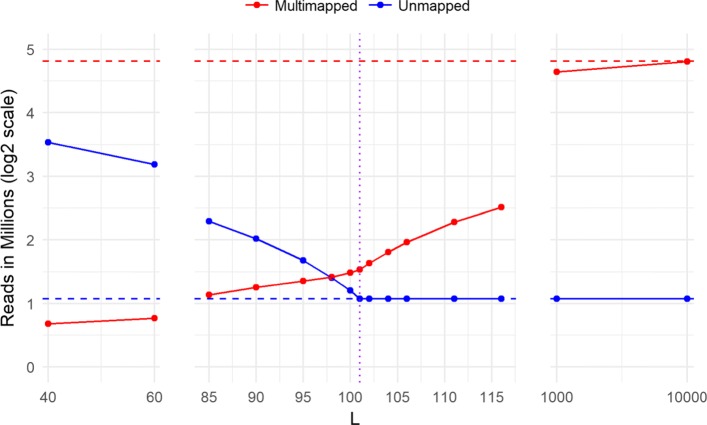
Alignment performance using segments from human transcriptome, tested for different values of *L*, to align 40 million reads of length 101 (first sample in SwitchTx dataset, see section 3). Performance is shown in terms of the number of multimapped reads (red solid line) and unmapped reads (blue solid line), compared to the number of multimapped reads (red dotted line) and unmapped reads (blue dotted line) when aligning using the transcriptome

To study the impact of using the segments library instead of the transcriptome for alignment, we created segments library with different values of *L* and compared the number of multi-mapped and unmapped reads for each case to alignemnt to the full transcriptome. We used RapMap [6] as our k-mer based aligner, to align samples of 40 million simulated reads of length 101 (samples from the switchTx human dataset discussed in “Simulation Datasets” section) in a single-end mode. We tested values of *L* centered around *L*=101 with many values close to 101, in order to test how sensitive the results are to small changes in the selection of *L*. Figure 2 shows the alignment performance in terms of the number of multi-mapped reads (red solid line) and unmapped reads (blue solid line), compared to the number of multi-mapped reads (red dotted line) and unmapped reads (blue dotted line) when aligning using the transcriptome. Using segments highly reduces the number of multi-mapped reads produced mainly from reads mapped to a single genomic location but different transcripts. The plot shows that too short segments compared to the read length results in a lot of unmapped reads, while using long segments compared to the read length causes an increasing number of multimappings. Consequently, choosing *L* to be close to the read length is the optimal choice to minimize multimappings while maintaining a steady number of mapped reads. This significant reduction in multimappings reported from the alignment step eliminates the need for a quantification step to resolve the ambiguity when producing raw pseudo-alignment counts. It is important to note that the best segments configuration still produces some multimappings. These result from reads sequenced from paralogs and sequence repeats which are not handled by the current version of Yanagi. Nevertheless, using segments can achieve around 10-fold decrease in the number of multimappings.

#### The importance of maximality property

Yanagi generates maximal segments, as mentioned in Definition 4 (“Segments Properties” section), which are extended as much as possible between branching points in the segments graph. The purpose of this property is to maintain stability in the produced segment counts since shorter segments will inherently produce lower counts which introduces higher variability that can complicate downstream analysis. To examine the effect of the maximal property, we simulated 10 replicates from 1000 random genes (with more than two isoforms) from the human transcriptome using Ployester [14]. Additional file 1: Figure S3 shows the distribution of the coefficient of variation (CV) of the produced segment counts from segments with and without the maximal property. When segments are created without maximal property, the scatter plot clearly shows that maximal segments have lower CVs to their corresponding short segments for a majority of points (40% of the points has a difference in CVs >0.05). That corresponds to generating counts with lower means and/or higher variances if the maximal property was not enforced.

### Segment-based Gene Expression Analysis

We propose a segment-based approach to gene expression analysis to take advantage of pseudo-alignment while avoiding a transcript quantification step. The standard RNA-seq pipeline for gene expression analysis depends on performing k-mer based alignment over the transcriptome to obtain transcripts abundances, e.g. Transcripts Per Million (TPM). Then depending on the objective of the differential analysis, an appropriate hypothesis test is used to detect genes that are differentially expressed. Methods that perform differential gene expression (DGE) prepare gene abundances by summing the underlying transcript abundances. Consequently, DGE methods aims at testing for differences in the overall gene expression. Among these methods are: DESeq2 [15] and edgeR [16]. Such methods fail to detect cases where some transcripts switch usage levels while the total gene abundance is not significantly changing. Note that estimating gene abundances by summing counts from the underlying transcripts can be problematic, as discussed in [17]. RATs [18] on the other hand is among those methods that target to capture such behavior and tests for differential transcript usage (DTU). Regardless of the testing objective, both tests entirely depend on the transcript abundances that were obtained from algorithms like EM during the quantification step to resolve the ambiguity of the multi-mapped reads, which requires bias-correction modeling [8] adding another layer of complexity to achieve the final goal of gene-level analysis.

Our segment-based approach aims at breaking the coupling between the quantification, bias modeling, and gene expression analysis, while maintaining the advantage of using ultra-fast pseudo-alignment techniques provided by k-mer based aligners. When aligning over the L-disjoint segments, the problem of multimapping across target sequences is eliminated making the quantification step unecessary. Statistical analysis for differences across conditions of interest is performed on segment counts matrix instead of TPMs.

#### Kallisto’s TCC-based approach

Yi et al. introduce a comparable approach in [19]. This approach uses an intermediate set defined in Kallisto’s index core as equivalence classes (EC). Specifically, a set of k-mers are grouped into a single EC if the k-mers belong to the same set of transcripts during the transcriptome reference indexing step. Then during the alignment step Kallisto derives a count statistic for each EC. The statistics are referred to as Transcript Compatibility Counts (TCC). In other words, Kallisto produces one TCC per EC representing number of fragments that appeared compatible with the corresponding set of transcripts during the pseudo-alignment step. Then the work in [19] uses these TCCs to directly perform gene-level differential analysis by skipping the quantification step using logistic regression and compared it to other approaches like using DESeq2. We will refer to that direction as the TCC-based approach. To put that approach into perspective with our segment-based approach, we will discuss how the two approaches compare to each other.

#### Comparison between segment-based and TCC-based approaches

**Fig. 3:**
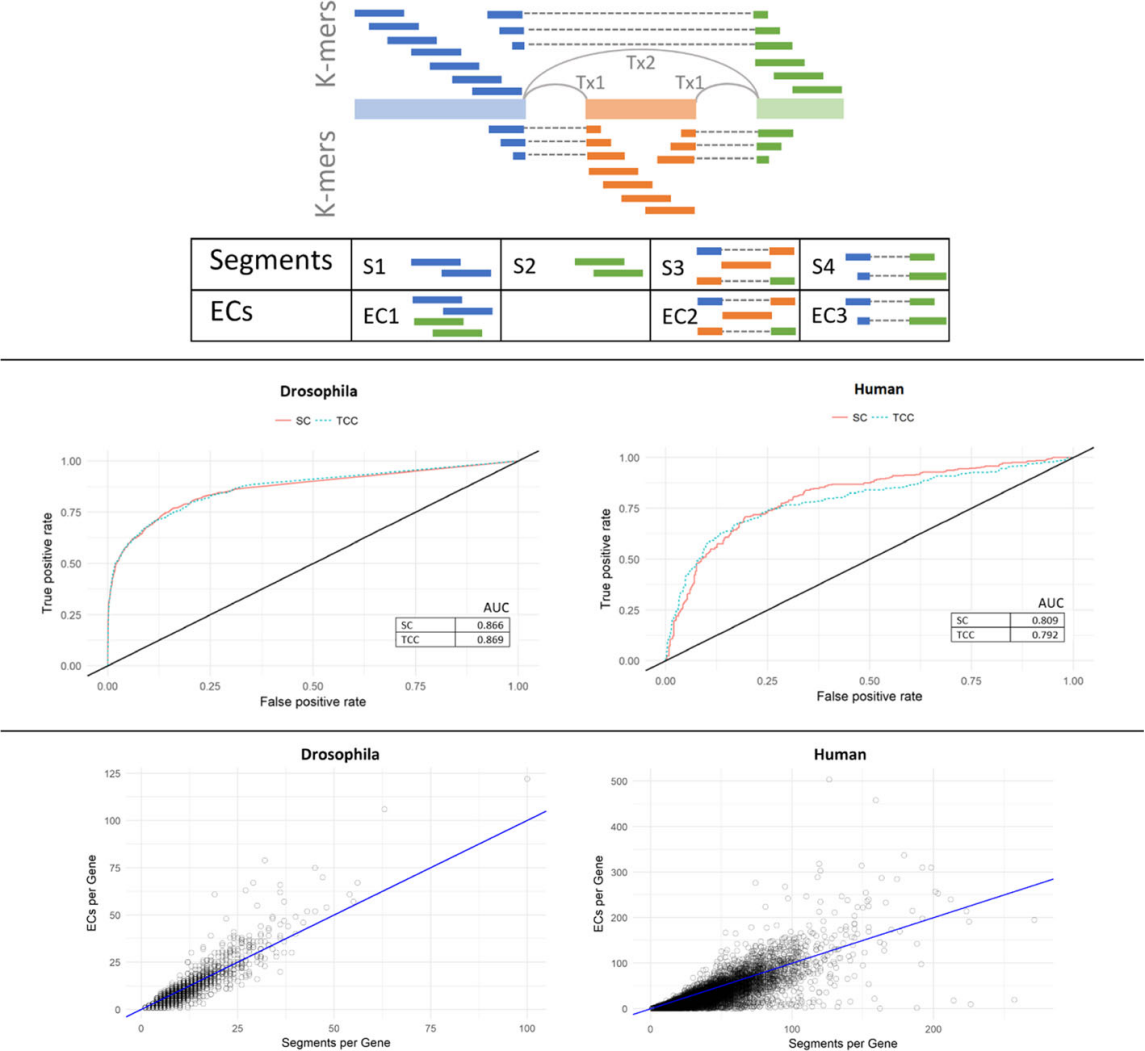
Segment-based gene-level differential expression analysis. (**Top**) Diagram showing an example of two transcripts splicing three exons and their corresponding segments from Yanagi versus equivelance classes (ECs) from kallisto. K-mer contigs from the first and last exons are merged into one EC (EC1) in kallisto while Yanagi creates two segments, one for each exon (S1, S2), hence preserving their respective location information. Both Kallisto and Yanagi generate ECs or segments corresponding to exon inclusion (EC2, S3) and skipping (EC3, S4). (**Middle**) ROC curve for simulation data for DEX-Seq based differential gene-level differential expression test based on segment counts (SC) and Kallisto equivalence class counts (TCC) for *D. melanogaster* and *H. sapiens*. (**Bottom**) Scatter plot of number of segments per gene (x-axis) vs. Kallisto equivalence classes per gene (y-axis) for the same pair of transcriptomes

Both segment-based and TCC-based approaches avoid a quantification step when targeting gene-level analysis. This can be seen as an advantage in efficiency, speed, simplicity, and accuracy, as previously discussed. One difference is that segment-based approach is agnostic to the alignment technique used, while TCC-based approach is a Kallisto-specific approach. More importantly, statistics derived in segment-based approach are easily interpretable. Since segments are formed to preserve the genomic location and splicing structure of genes, Segment Counts (SC)s can be directly mapped and interpreted with respect to the genome coordinates. In contrast, ECs do not have a direct intepretation in this sense. For instance, all k-mers that belong to the same transcript yet originated from distinct locations over the genome will all fall under the same EC, making TCCs less interpretable. Figure 3-top shows a toy example for a simple case with two transcripts and three exons along with its resulting segments and ECs. In this case, k-mer contigs from the first and last exons are merged into one EC (EC1) in Kallisto, while Yanagi creates a separate segment for each of the two constitutive exons (S1, S2), hence preserving their respective location information. This advantage can be crucial for a biologist who tries to interpret the outcome of the differential analysis. In the next section we show a segment-based gene visualization that exploits the genomic location information of segments to allow users to visually examine what transcripts exons and splicing events contributed to differences for genes identified as determined differentially expressed.

Figure 3-bottom shows the number of Yanagi’s segments per gene versus the number of Kallisto’s equivalence classes per gene. The number of equivalence classes were obtained by building Kallisto’s index on human transcriptome, then running the pseudo command of Kallisto (Kallisto 0.43) on the 6 simulated samples from SwitchTx dataset (“Simulation Datasets” section).

Note that, in principle there should be more segments than ECs since segments preserve genome localization, however in practice Kallisto reports more ECs than those discovered in the annotation alone in some genes. The extra ECs are formed during pseudo-alignment when reads show evidence of unannotated junctions.

#### DEXSeq-based model for differential analysis

In this work we adopt the DEXSeq [20] method to perform segment-based gene differential analysis. DEXSeq is a method that performs differential exon usage (DEU). The standard DEXSeq workflow begins by aligning reads to a reference genome (not to the transcriptome) using TopHat2 or STAR [21] to derive exon counts. Then, given the exon counts matrix and the transcriptome annotation, DEXSeq tests for DEU after handling coverage biases, technical and biological variations. It fits, per gene, a negative binomial (NB) generalized linear model (GLM) accounting for effect of the condition factor, and compares it to the null model (without the condition factor) using a chi-square test. Exons that have their null hypotheses rejected are identified as differentially expressed across conditions. DEXSeq can tehn produce a list of genes with at least one exon with significant differential usage and controls the false discovery rate (FDR) at the gene level using the Benjamini–Hochberg procedure.

We adopt the DEXSeq model for the case of segments by replacing exons counts with segments counts, the latter derived from pseudo-alignment. Once segments are tested for differential usage across conditions, the same procedure provided by DEXSeq is used to control FDR on the list of genes that showed at least one segment with significant differential usage.

We tested that model on simulated data (SwitchTx dataset in “Simulation Datasets” section) for both human and fruit fly samples and compared our segment-based approach with the TCC-based approach since they are closely comparable. Since the subject of study is the effectiveness of using either SCs or TCCs as a statistic, we fed TCCs reported by Kallisto to DEXSeq’s model as well to eliminate any performance bias due the testing model. As expected, Fig. 3-middle shows that both approaches provide highly comparable results on the tested dataset. Recall that using segment counts to test for differentially expressed genes adds to the interpretability of the test outcomes.

Although that experiment was chosen to test the use of SCs or TCCs as statistics to perform differential usage, different gene-level tests can also be performed on segment counts. For instance, testing for significant differences in overall gene expression is possible based on segment counts as well. A possible procedure for that purpose would be using DESeq2. One can prepare the abundance matrix by R package tximport [22], except that the matrix now represent segment instead of transcript abundances. The next section shows how visualizing segment counts connects the result of some hypotheses testing with the underlying biology of the gene.

### Segment-based Gene Visualization

**Fig. 4:**
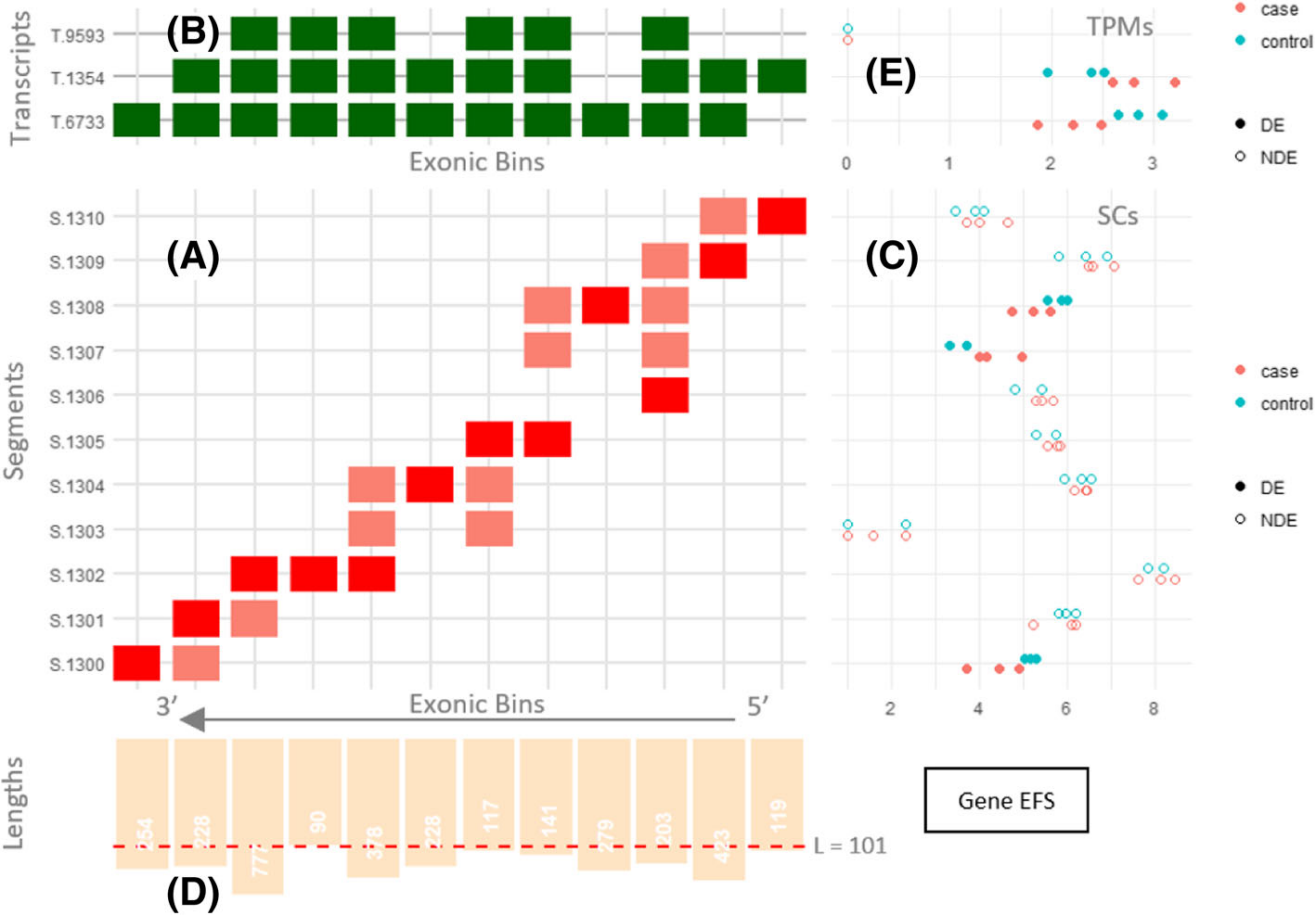
Visualizing segments and segment counts of a single gene with differentially expressed transcripts. It shows human gene EFS (Ensembl ENSG00000100842). The gene is on the reverse strand, so the bins axis is reversed and segments are created from right to left. (**a**) Segment-exonic bin membership matrix, (**b**) Transcript-exonic bin membership matrix. (**c**) Segment counts for three control and three case samples, fill used to indicate segments that were significantly differential in the gene. (**d**) Segment length bar chart, (**e**) (optional) Estimated TPMs for each transcript

Figure 4 shows Yanagi’s proposed method to visualize segments and the segment counts of a single gene. The plot includes multiple panels, each showing a different aspect of the mechanisms involved in differential expression calls. The main panel of the plot is the segment-exon membership matrix (Panel A). This matrix shows the structure of the segments (rows) over the exonic bins (columns) prepared during the annotation preprocessing step. An exon (or a retained intron) in the genome can be represented with more than one exonic bin in case of within-exon splicing events (See Step 1 in “Segmentation Algorithm” section). Panel B is a transcript-exon membership matrix. It encapsulates the transcriptome annotation with transcripts as rows and the exonic bins as columns. Both membership matrices together allow the user to map segments (through exonic bins) to transcripts.

Panel C shows the segment counts (SCs) for each segment row. Panel D shows the length distribution of the exonic bins. Panel E is optional. It adds the transcript abundances of the samples, if provided. This can be useful to capture cases where coverage biases over the transcriptome is considered, or to capture local switching in abundances that are inconsistent with the overall abundances of the transcripts. The exonic bins axis is reversed and segments are created from right to left as the gene shown is on the reverse strand.

Consider the top-most segment (S.1310) for instance. It was formed by spanning the first exonic bin (right-most bin) plus the junction between the first two bins. This junction is present only at the second transcript (T.1354) and hence that segment belongs to only that transcript. In the segment-exon matrix, red-colored cells mean that the segment spans the entire bin, while salmon-colored cells represent partial bin spanning; usually at the start or end of a segment with correspondence to some junction.

Alternative splicing events can be easily visualized from Fig. 4. For instance, the third and fourth segments from the top (S.1308 and S.1307) represent an exon-skipping event where the exon is spliced in T.6733 and skipped in both T.1354 and T.9593.

### Segment-based Alternative Splicing Analysis

The analysis of how certain genomic regions in a gene are alternatively spliced into different isoforms is related to the study of relative transcript abundances. For instance, an exon cassette event (exon skipping) describes either including or excluding an exon between the upstream and downstream exons. Consequently, isoforms are formed through a sequential combination of local splicing events. For binary events, the relative abundance of an event is commonly described in terms of percent spliced-in (PSI) [23] which measures the proportion of reads sequenced from one splicing possibility versus the alternative splicing possibility, while *ΔPSI* describes the difference in PSI across experimental conditions of interest.

Several approaches were introduced to study alternative splicing and its impact in studying multiple diseases. [24] surveyed eight different approaches that are commonly used in the area. These approaches can be roughly categorized into two categories depending on how the event abundance is derived for the analysis. The first category is considered count-based where the approach focuses on local measures spanning specific counting bins (e.g. exons or junctions) defining the event, like DEXSeq [20], MATS [25] and MAJIQ [26]. Unfortunately, many of these approaches can be expensive in terms of computation and/or storage requirements since it requires mapping reads to the genome and subsequent processing of the large matrix of counting bins. The second category is isoform-based where the approach uses the relative transcript abundances as basis to derive PSI values. This direction utilizes the transcript abundance (e.g. TPMs) as a summary of the behavior of the underlying local events. Cufflinks [4, 17], DiffSplice [27] and SUPPA [28, 29] are of that category. Unlike Cufflinks and DiffSplice which perform read assembly and discovers novel events, SUPPA succeeds in overcoming the computational and storage limitations by using transcript abundances that were rapidly prepared by lightweight k-mer counting alignment like Kallisto or Salmon.

One drawback of SUPPA and other transcript-based approaches alike is that it assumes a homogeneous abundance behavior across the transcript making it susceptible to coverage biases. Previous work showed that RNA-seq data suffers from coverage bias that needs to be modeled into methods that estimate transcript abundances [30, 31]. Sources of bias can vary between fragment length, positional bias due to RNA degradation, and GC content in the fragment sequences.

**Fig. 5:**
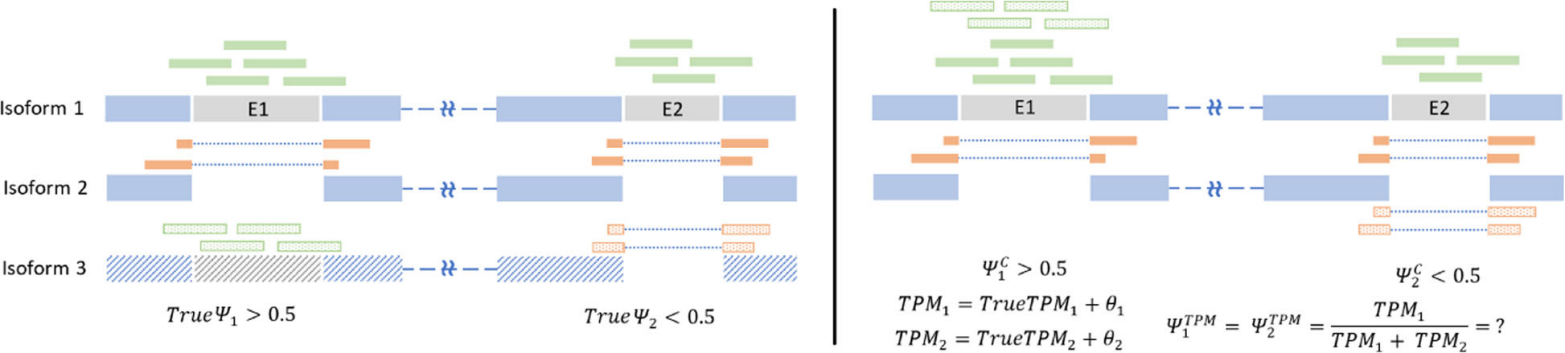
This diagram illustrates a problem with transcript-based approaches for calculating *PSI* in the presence of unannotated transcripts. (**Left**) shows the truth, with three isoforms combining two exon skipping events (E1, E2). However, isoform 3 is missing from the annotation. Reads spanning both events are shown along their true source. Reads spanning an exon incluion are colored green whereas reads spanning a skipping junction are colored orange. (**Right**) shows the problem with *PSI* values from transcript abundance. Because these two alternative splicing events are coupled in the annotation, their *PSI* values calculated from transcript abundances will always be the same ($\psi ^{TPM}_{1}$ = $\psi ^{TPM}_{2}$), even though the true values are not (*Trueψ*_1_≠*Trueψ*_2_). Furthermore, changes in the estimated abundances (*TPM*_1_,*TPM*_2_) make the calculated *PSI* values unpredictable. Count-based *PSI* values ($\psi ^{C}_{1}, \psi ^{C}_{2}$) on the other hand correctly reflect the truth

Another critical drawback with transcript-based approaches is that its accuracy highly depends on the completeness of the transcript annotation. As mentioned earlier standard transcriptome annotations enumerate only a parsimonious subset of all possible sequential combinations of the present splicing events. Consider the diagram in Fig. 5 with a case of two annotated isoforms (Isoform 1 and 2) whereas a third isoform (isoform 3) is missing from the annotation. The three isoforms represent three possible combinations of two splicing events (skipping exons E1 and E2). If the two events are sufficiently far apart in genomic location, short reads would fail to provide evidence of the presence of isoform 3, leading to mis-assignment of reads into the other two isoforms (Fig. 5 right). That behavior can bias the calculated *PSI* values of both events E1 and E2. Even if the mis-assigned reads did not change the estimation of *TPM*_1_ and *TPM*_2_, the calculated *PSI*s for both events can be significantly far from the truth. Further in this paper we refer to any pair of events that involves such behavior as coupled events.

Our segment-based approach works as a middle ground between count-based and transcript-based approaches. It provides local measures of splicing events while avoiding the computational and storage expenses of count-based approaches by using the rapid lightweight alignment strategies that transcript-based approaches use. Once the segment counts are prepared from the alignment step, Yanagi maps splicing events to their corresponding segments, e.g. each event is mapped into two sets of segments: The first set spans the inclusion splice, and the second for the alternative splice (See “Segment-based calculation of PSI” section). Current version of Yanagi follows SUPPA’s notation for defining a splice event and can process seven event types: Skipped Exon (SE), Retained Intron (RI), Mutually Exclusive Exons (MX), Alternative 5’ Splice-Site (A5), Alternative 3’ Splice-Site (A3), Alternative First Exon (AF) and Alternative Last Exon (AL).

### Comparing Segment-based and isoform-based PSI values with incomplete annotation

To show how the estimated transcript abundances in the case of incomplete annotations can affect local splicing analysis, we ran both SUPPA and Yanagi pipelines on dataset simulating situations like the one in Fig. 5. We simulated reads from 2454 genes of the human genome. A novel isoform is formed in each gene by combining two genomically distant events in the same gene (coupled events) where the inclusion of the first and the alternative splicing of the second does not appear in any of the annotated isoforms of that gene (IncompTx dataset in “Simulation Datasets” section). After reads are simulated from the annotated plus novel isoforms, both SUPPA and Yanagi pipelines where run with the original annotation which does not contain the novel isoforms.

**Fig. 6:**
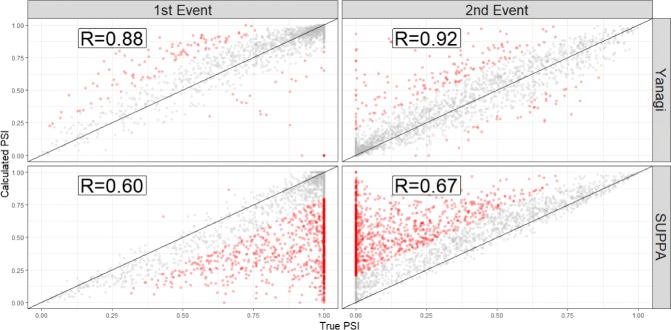
The PSI values of 2454 coupled events formulating novel isoforms used in simulated data to simulate scenarios of incomplete annotation, similar to Fig. 5. Each novel isoform consists of combining the inclusion splicing of the first event and the alternative (skipping) splicing of the second event. PSI values obtained by Yanagi and SUPPA are compared to the true PSI values. Red points are measures of error larger than 0.2. SUPPA tends to underestimate the PSI of the first event and overestimate in the second event (43% of the points are red compared to only 7% in Yanagi)

Figure 6 shows the calculated PSI values of the coupled events compared to the true PSI values. It is clear how the PSI values for both events can be severely affected by the biased estimated abundances. In SUPPA’s case, abundance of both sets of inclusion and exclusion isoforms were overestimated. However, the error in abundance estimates of inclusion transcripts were consistently higher than the error in exclusion transcripts. Therefore, the PSI values of the second event were consistently overestimated by SUPPA whereas PSI values of the first events were consistently underestimated. Furthermore, splicing events involving the affected isoforms will be inherently affected as well even when they were unrelated to the missing transcript. This coupling problem between events inherent in transcript-based approaches is circumvented in values calculated by Yanagi, and generally, by count-based approaches.

**Fig. 7:**
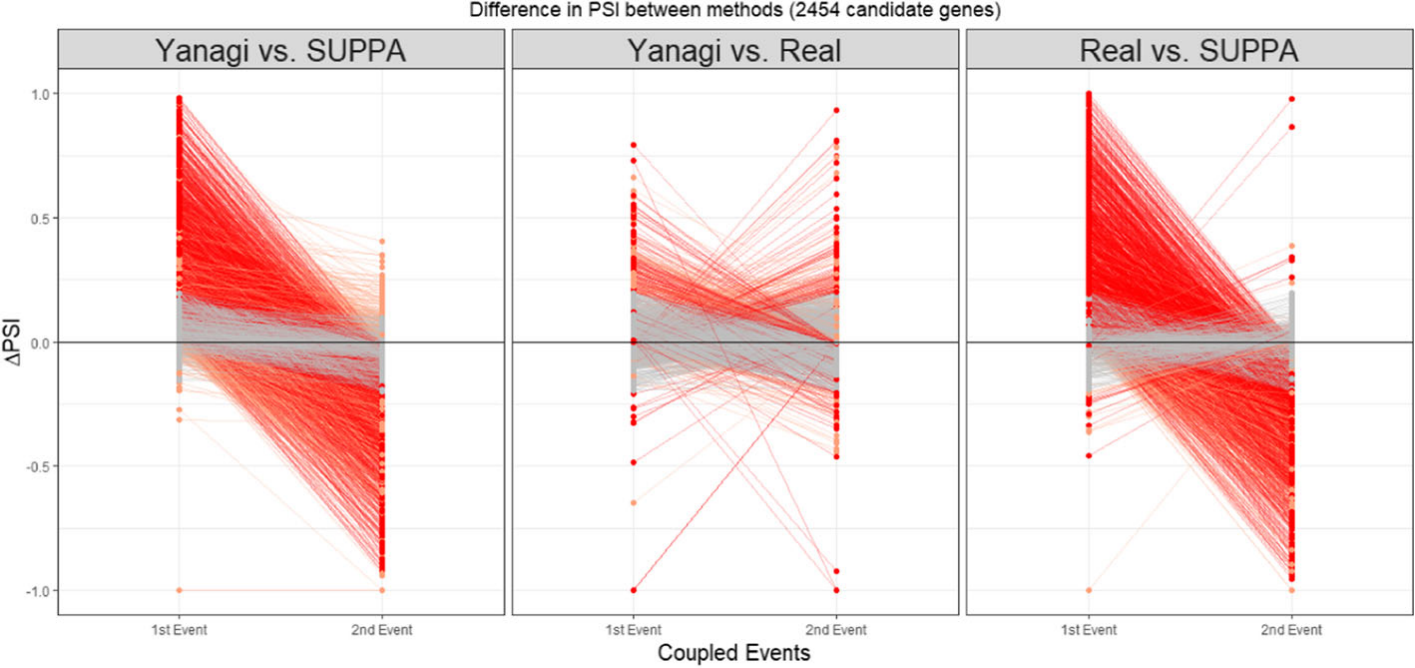
Trends of error in event PSI values across methods. *ΔPSI* of an event is calculated here as the difference in the calculated *PSI* of that event obtained either by Yanagi, SUPPA, or the truth. For each coupled event, a line connecting *ΔPSI* of the first event to the second’s is drawn to show the trend of change in error among the first and second event in each pair. Overestimation-to-underestimation (and underestimation-to-overestimation) trends are colored red. Orange colored trends represent trends where both events were either overestimated or underestimated. Trends with insignificant differences (|*ΔPSI*|<0.2) are colored grey

Figure 7 shows the trends in estimation error of *PSI* across methods for the 2454 coupled events. *ΔPSI* of an event is calculated here as the difference between the calculated *PSI* of that event obtained either by Yanagi or SUPPA, and the true *PSI*. For each splicing event couple, a line connecting *ΔPSI* of the first event to the second’s is drawn to show the trend of change in error between the first and second event in each pair. We found that estimates by SUPPA drastically exhibit a trend we refer to as overestimation-to-underestimation (or underestimation-to-overestimation) in 50% of the pairs while 36% of the pairs showed minor errors (*ΔPSI*<0.2). Yanagi’s estimates on the other hand showed the further trend only in 7% of the pairs while 87% of the pairs showed minor errors.

### Comparing Segment-based and isoform-based PSI values on drosophila melanogaster

Based on known complexity and incompleteness of the *D*rosophila melanogaster transcript annotation we examined an RNA-seq dataset of male fly head (available online with GEO accession number GSM2108304) for evidence of similar behavior to that studied in the previous simulation. Since the true *PSI* values are unknown, we compare the trends of the difference in *PSI* between SUPPA and Yanagi. We add to the comparison the *PSI*s obtained from a count-based approach, rMATS.

The scenario studied in the simulation is just one possible scenario of missing isoforms. More complex scenarios are likely to occur in real situations. Complex scenarios may include missing more than one isoform or when the event coupling problem involves more than two events. Such scenarios make detecting the full scale of the problem more complicated. Here we focus on the issue of coupled events as described in our simulation.

**Fig. 8:**
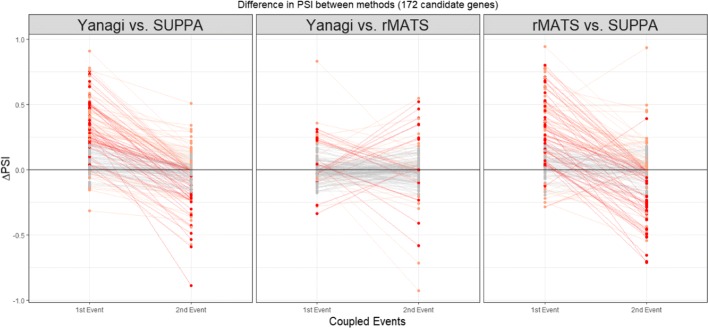
Trends in *ΔPSI* across methods Yanagi, SUPPA, rMATS for 172 coupled events in candidate genes for incomplete annotation in drosophila melanogaster (SRR3332174). Overestimation-to-underestimation (and underestimation-to-overestimation) trends are colored red. Orange colored trends represent trends where both events were either overestimated or underestimated. Trends with insignificant differences (|*ΔPSI*|<0.2) are colored grey. Out of the 172 cases, 33% showed Overestimation-to-underestimation (or underestimation-to-overestimation) trends in Yanagi-SUPPA, 11% in Yanagi-rMATS, 29% in rMATS-SUPPA

We follow the same analogy used in the simulation to define coupled events and find candidate genes of at least one missing isoform that couples two sufficiently distant events. By searching genes only in the forward strand and only events of type SE, A3, A5, we found 172 candidate genes and pair of coupled events where some splicing combination is possibly missing. Note that this candidate search is independent of the RNA-seq data, or the segment generation process. Figure 8 shows the trends in *ΔPSI* between Yanagi, SUPPA and rMATS for the 172 cases of coupled events. Evidence of overestimation-to-underestimation trends were found between SUPPA and both Yanagi and rMATS, suggesting a similar behavior to the phenomenon present in our simulation (33% in Yanagi-SUPPA, 11% in Yanagi-rMATS, 29% in rMATS-SUPPA). It should be noted that those 172 cases of coupled events were only selected from part of the genome as candidates of one scenario of missing isoforms which means it is very likely for more cases to exist at the scale of the whole transcriptome. Additional file 1: Figure S4 shows a scatter plot of the *PSI* values of full list of events found in the transcriptome annotation.

**Fig. 9:**
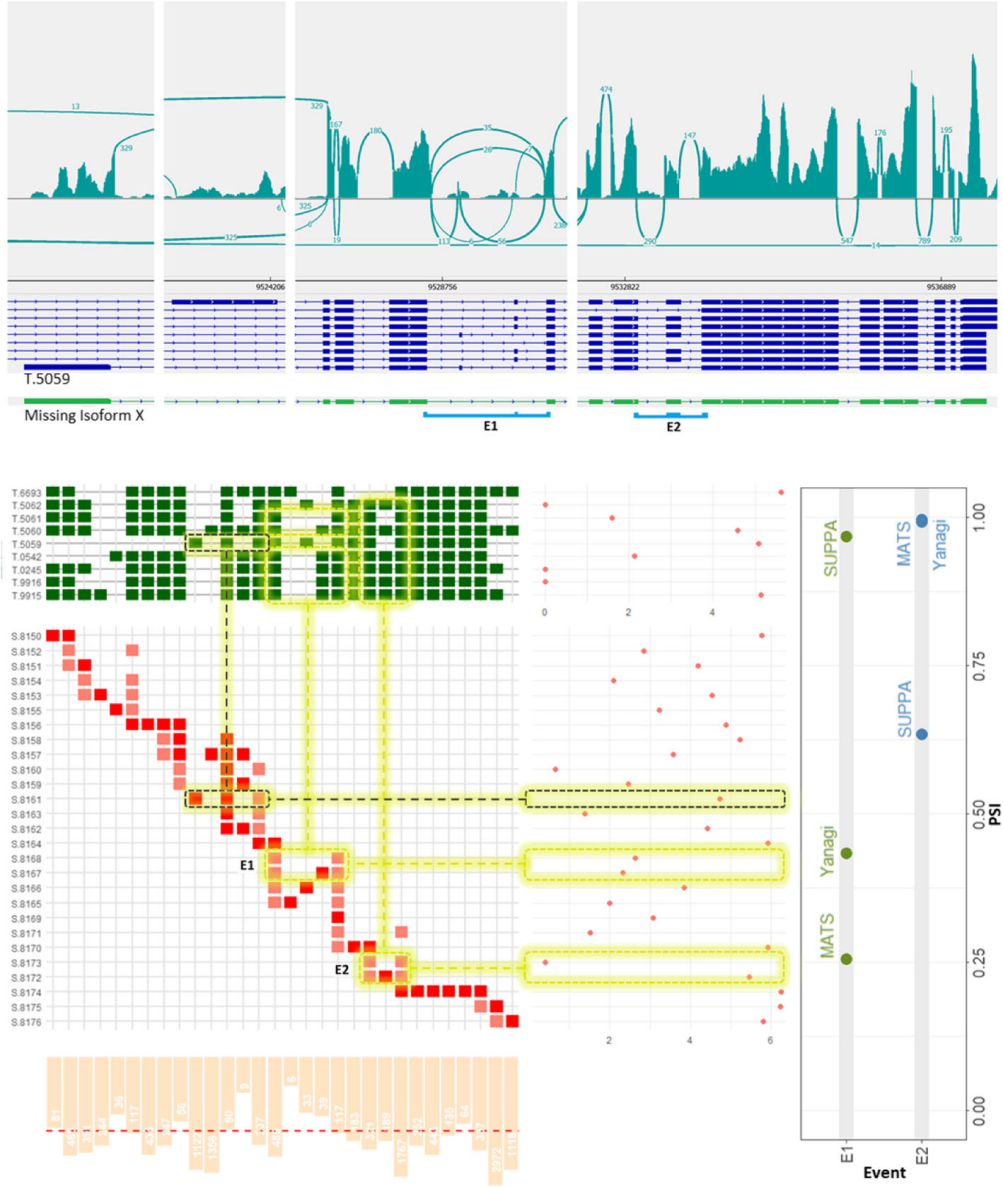
The *Bruchpilot* gene in *Drosophila melanogaster* (SRR3332174) serves as an example of a gene likely to have incomplete annotation. (**Bottom-Right**) The *PSI* values of the coupled events E1 and E2 exhibit severe overestimation and underestimation, respectively, by transcript-based approaches compared to Yanagi and rMATS. (**Top**) illustrates read coverage across the gene prepared using IGV, aligned with the 9 annotated isoforms. (**Bottom-Left**) The segments visualization of the gene is compared to transcript-level expression (TPM) obtained from kallisto, and the segment counts (normalized) from Yanagi’s pipeline. Refer to section 3 for details on this panel’s components. Postulating a isoform X (shown as a green-colored track on the top panel) missing from the annotation explains the deviation in both *PSI* values and the inconsistency in coverage across transcript T.5059

We study the Bruchpilot gene (FBgn0259246) as a specific illustration of a candidate gene with coupled events exhibiting overestimation-to-underestimation trend in SUPPA’s *ΔPSI*s on Drosophila sample SRR3332174. Figure 9 shows three panels: (top panel) the read coverage of the genomic region of the gene by IGV alongside the 9 annotated transcripts, (bottom left panel) the segments visualization and its counts along with the transcripts abundances estimated by Kallisto, (bottom right panel) the *PSI* values of the coupled events E1, E2 calculated by SUPPA, Yanagi and rMATS. The read coverage for both events supports Yanagi’s results rather than SUPPA’s. The overestimation of one particular transcript, NM_001259298.2 (T.5059 in figure), can be one potential cause of such deviation. As the read coverage panel shows, most of the reads supporting that transcript are in fact coming from the first coding exon (its junction segment is highlighted grey) whereas the rest of the junctions, e.g. the skipping junction in E1, does not show sufficient coverage supporting its high abundance estimated by Kallisto. One possible explanation is that the annotation is missing isoform X (colored green on the top panel). It is the same as the present transcript T.5059 except it combines the skipping splicing for E1 and the inclusion splicing for E2. The inclusion of isoform X in the annotation during transcript abundance estimation would have directed most reads aligned to the first exon towards isoform X rather than T.5059 for a more consistent coverage over both transcripts. Consequently, SUPPA’s *PSI* values for both E1 and E2 would align better with Yanagi and rMATS values.

### Comparing segment-based PSI values with counting-based and isoform-based PSI values

Here we are comparing PSI values obtained from Yanagi (See “Segment-based calculation of PSI” section) versus counting-based approaches like rMATS and isoform-based approaches like SUPPA on a very controlled setting. In that setting, we expect no significant difference between measures obtained from each of the three approaches. We used the simulation of switching abundance dataset (SwitchTx dataset in “Simulation Datasets” section). Since each tool provides separate set of events, we focus our comparison on the intersection set of events between SUPPA and rMATS. That includes events from five types of splicing events. Table 2 summarizes the number of events subject to the study. Two levels of filtering are applied to observe how the different approaches behave in different scenarios. Non-overlapping events is the smallest subset of events. Those events exclude complex splicings where more than two splicings define the event. While highTPM events is a subset of events in which inclusion and exclusion isoform levels are relatively high (*TPM*_*inc*_>1,*TPM*_*ex*_>1). This is a typical filtering criterion adopted by isoform-based approaches. This filter excludes events involving isoforms of low levels of expression which inherently suffer from low estimation accuracy. Note that when complex events are included, they are treated as a set of separate binary events.

**Table 2:** Running time (seconds) and memory usage (gigabytes) by Yanagi to generate segment library for fruit fly (BDGP6) and human (GRCh38) genomes, for both the preprocessing and segmentation steps

	BDGP6			GRCh38	
	time(s)	memory(GB)		time(s)	memory(GB)
Preprocessing	13	0.9		112	1.5
Segmentation					
L=40	20	0.4		248	1.3
L=108	20	0.4		250	1.3
L=1000	20	0.4		228	1.3
L=10000	8.5	0.4		77	1.3
Rapmap Indexing (4 Threads)					
L=108	103	0.8		420	2.6
Txs	121	1.1		480	3.7
Rapmap Quantification (8 Threads)					
L=108	236	0.7		220	2.1
Txs	292	1.2		416	3.1

Time for the preprocessing step does not include the time to load the FASTA and GTF files. Most of the memory usage is from loading the input data in both steps. Running on a 6-core 2.1 GHz AMD processor, using single-threaded processes. The lower half shows the time and memory usage for running Rapmap’s quasi-mapping using the segments library and the the full transcriptome, to quantify samples of 40M paired-end reads, each of length 101bp.

**Fig. 10:**
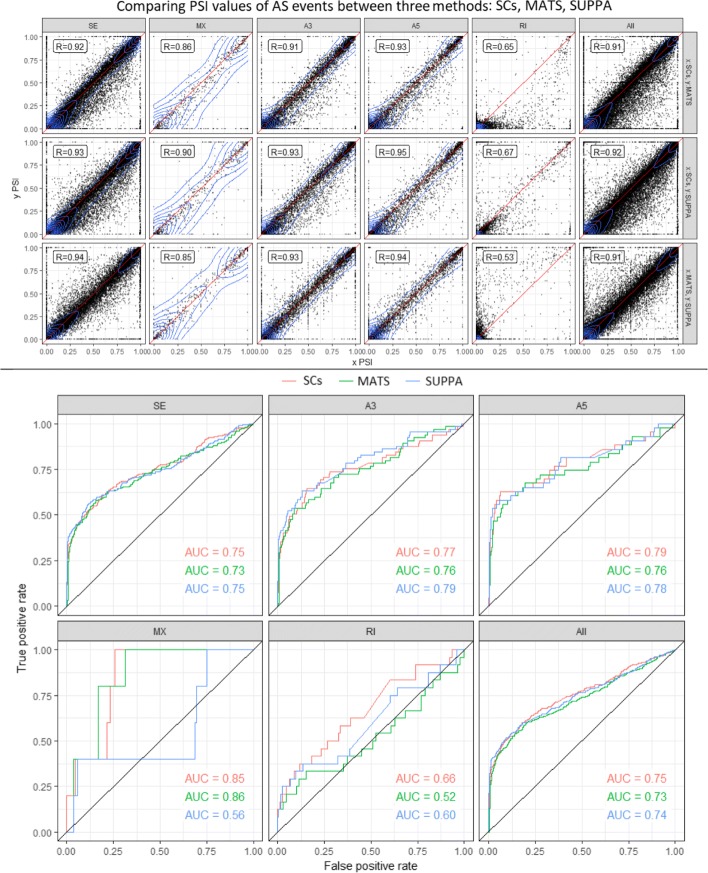
(**Top**) Comparing PSI values calculated using segment counts versus rMATS (first row), segment counts vs SUPPA (second row) and rMATS versus SUPPA (third row) on human samples from SwitchTx simulated dataset. Columns indicate seven types of alternative splicing events. (**Bottom**) Comparing ROC curves for differential alternative splicing using segment counts, rMATS and SUPPA for simulation dataset of switched abundance. Plots are stratified by event types. See Table 2 for number of events of each AS event type shown

Figure 10 (Top) shows a scatter plot of PSI values calculated by the three approaches for all events. Separate plots for the filtered events in Additional file 1: Figure S5. Among the five different splicing types exon skipping, alternative 3’ and alternative 5’ events give the highest correlation between segment counts and rMATS approaches. In our experiments we noticed that rMATS (v4.0.1) does not behave as intended for intron retention events. We noticed that counts including junction reads only and counts including both junction and intron reads (which we use in this study) are the same. In other words, rMATS fails to report reads spanning the intron, which explains the underestimated inclusion counts and PSI values for retained introns.

It should be noted that most count-based approaches require aligning to the genome which is usually the bottle-neck process in the pipeline that some try to overcome in the expense of storage by storing large intermediate data (BAM files). The major motivation of transcript-based approaches is to achieve fast and light-weight pipelines that is not that expensive in terms of time and memory. For instance, even when using STAR, which is one of the fastest genome mappers in the field, using pseudo-alignment tools can be several orders of magnitude faster (or efficient in terms of storage and memory). That is why our segments approach is unique in leveraging such light-weight tools that utilizes pseudo-alignment algorithms with the capability of obtaining local measurements.

#### Segment-based Differential Alternative Splicing

Since the scope of this paper is to introduce the use of segment counts as a statistic for studying alternative splicing, we want to use the simplest statistical model for differential splicing to exclude any advantage obtained by the model itself. In that matter we used the PSI values of the three approaches (SCs, rMATS, SUPPA) as discussed in the previous section. Then we used a linear model for differential hypothesis testing (implemented with Limma-voom R Package [32, 33]). However, more advanced models of differential analysis can be used instead. For example, a similar model to SUPPA2 can be developed to test the significance of *ΔPSI* by considering all events genome-wide [29]. Figure 10 (Bottom) shows ROC plots for sensitivity and specificity measures. Using segment counts achieves comparable performance to both rMATS and isoform-based approaches in that setting.

## Discussion

Recent efforts like recount2 [34] and refine.bio [35] provide comprehensive uniformly processed summary data for large repositories of RNA-seq data. refine.bio uses psuedo-mapping procedures to process data and thus provide statistics at transcript level resulting from a transcript quantification step. This precludes the direct use of these data in downstream analyses where transcript quantification is not essential. Recount2 provides data as exon and junction-level counts but requires genome alignment procedures which are computationally heavier and prone to errors (e.g. in the case of extremely small exons). Our proposed segment approach provides a useful compromise for these large-scale uniform data catalogs between using lightweight pseudo-mapping and providing data directly usable in a variety of expression analyses.

Recent work done on alternative splicing, e.g. Whippet [36] and ASGAL [37], may seem similar to Yanagi’s approach since they all rely on processing the splice graph. ASGAL uses graph-based alignment approach to align reads directly into the splice graph which may introduce more complexity processing and traversing the graph. Whippet prepares and indexes what it defines as contiguous splice graph (CSG) before linear alignment of reads is performed. Both methods are built solely for the purpose of alternative splicing analysis. Yanagi’s motivation and objective is different. It is important to note that the intent of this work is not to propose another alternative splicing method, but rather to introduce a conceptual framework that extends pseudo-alignment techniques through decoupling the alignment and quantification steps to generate statistics suitable to a variety of downstream analyses, including alternative splicing.

Alternative Splicing (AS) methods that use transcript abundance, provided that a complete transcript annotation and a transcript quantification method that sufficiently addresses coverage bias across a transcript is used, can provide an advantage over methods that only use local information for AS analysis, including AS based on segment counts produced by Yanagi. Nonetheless, as we discussed elsewhere in the manuscript, there is no loss of information in segment counts and they may be used to perform transcript quantification or as statistics into a AS method that borrows information across splicing events to take advantage of their correlation.

This type of extension on the use of segment counts to perform transcript quantification is a fruitful direction for future research. Another interesting extension of our work would be to study the use of segments in discovering novel transcripts. Using paired-end reads mapped to two segments that do not share any common transcripts can be a potential direction.

For the moment, analysts using ultra-fast pseudo-mapping will need to decide if they prefer possible loss of performance in AS analysis from using only local information, or from using an incomplete annotation. We believe that the results we show in our paper are informative in this situation. In Section 2.6, we showed how severely an incomplete annotation can decrease the correlation of PSI estimates with the truth (0.6 compared to 0.9 when using segments). Incomplete annotations are common in species with multiple introns per gene because the standard is to report a parsimonious set of transcripts rather than a complete set that represents all combinations of local splicing choices. We also showed in Section 2.8 an analysis on simulated data where the annotation is complete comparing the performance of the segments approach to an approach that makes use of information from other parts of the transcript (SUPPA). We observed that segment-based PSIs, that didn’t use the information in the other parts of the transcript unlike transcript-based PSIs, obtain a 0.92 correlation with those PSI values estimated using that information. Given these results indicating there is greater loss of performance when using an incomplete annotation compared to the exclusive use of local information, we suggest that a conservative approach based on segment counts, which is more robust to incomplete annotation, is used for AS analysis.

The current version of Yanagi, discussed here, generates L-disjoint segments from gene independently, since that is arguably the major cause of ambiguity from multimapping reads. However, other sources of ambiguity (such as overlapping genes and paralogs) are also of interest. That can be tackled in future versions by processing multiple genes simultaneously in the segmentation step.

Here we have discussed the use of segments and segment counts in two resolutions of RNA-seq analysis: gene level expression estimates and local alternative splicing. We demonstrated comparable results while avoiding the transcript quantification step completely. A natural extension to our work is to study the use of segments into the middle resolution of transcript level analysis. We hypothesize that the usage of segments can simplify the task of transcript abundance estimation and enable simpler incorporation of different sources of bias. Consequently, downstream analyses where quantification is appropriate are still available after generating segment-level counts.

## Conclusions

In this paper we have formalized the concept of transcriptome segmentation and proposed an efficient algorithm for generating segment libraries from transcript libraries based on a length parameter *L* (typically chosen dependent on an experiment-specific RNA-seq library construction). The resulting segment sequences are used with pseudo-alignment tools to quantify expression at the segment level, providing sufficient information for a variety of expression analyses. We have characterized segment libraries for the reference transcriptomes of *Drosophila melanogaster* and *Homo sapiens* for various read-length RNA-seq experimental designs. We also provide a novel gene-level visualization of transcriptome segments and transcript structure for ease of interpretation. Finally, we have demonstrated the use of segment-level quantification in differential gene expression and alternative splicing analysis.

Using a segment library rather than the standard transcriptome succeeds in significantly reducing ambiguous alignments where reads are multi-mapped to several sequences in the reference, thereby decoupling the pseudo-alignment and quantification steps used in current k-mer based pipelines for gene expression analysis. Moreover, using segment counts as statistics for gene-level differential expression and alternative splicing analyses achieves performance comparable to counting-based approaches (e.g. rMATS for splicing analysis) while using fast and lightweight pseudo-alignment. The notion of transcript segmentation as introduced here and implemented in Yanagi has the potential to extend the application of lightweight, ultra-fast, pseudo-alignment algorithms to a wider variety of RNA-seq analyses.

## Methods

### Transcriptome Segmentation

Figure 1 shows a typical situation in RNA-seq data analysis and provides an overview of the transcript segmentation strategy. In particular, it summarizes how reads that would be multi-mapped when aligning to a transcript library would be aligned to segments. In the latter case, all reads are aligned to a single target sequence and read counts are obtained per segment without the need of probabilistic quantification methods to resolve ambiguity. The next few subsections present specifics of the Yanagi [38] method for transcriptome segmentation.

### Segments Properties

Yanagi’s objective is to generate a minimal set of disjoint sequences (where disjointness is parameterized by *L*, which is typically chosen to be the experimental sequencing read length), while maintaining transcriptome sequence completeness.

The following definitions are for a given transcriptome T, and parameter L.

#### **Definition 1**

(A Segment) A segment *seg* defined by the tuple 〈*exs,loc,w*〉 is a genomic region of width *w* beginning at genomic location *loc* and spanning the sequence of consecutive exonic regions *exs*∈*Exs*_*T*_ (either exons or retained introns). Exonic regions are considered consecutive if they are consecutively spliced into at least one possible isoform in T. And for all segments in a segment library *S*_*T,L*_, its width *w* is at least *L* bases.

#### **Definition 2**

(Segments Sequences Completeness) The set of segments *S*_*T,L*_ is *Complete* if and only if

$$\begin{aligned} seq \in S_{T, L}; \forall seq \in & {Substring}({T}), len(seq) \leq L\\ &\text{and}\\ seq \in {Substring}({T}); &\forall seq \in {Substring}(S_{T, L}) \end{aligned} $$

#### **Definition 3**

(L-disjoint Segments) Each segment in the set *S*_*T,L*_ is *L-disjoint* if and only if *width*[*overlap*(*seg*_*i*_,*seg*_*j*_)]<*L*;∀*seg*_*i*_,*seg*_*j*_∈*S,i*≠*j*

The L-disjointness property restricts any pair of *L-disjoint* segments to have an overlap region shorter than parameter *L*, which typically equals to the sequencing read length. In other words, no read of length at least *L* can be mapped to both segments of an *L-disjoint* segment pair, assuming error-free reads.

Another property of the generated segments is to be maximal. For *seg*:〈*exs,loc,w*〉, denote *Txs*(*seg*) as the set intersection of annotated transcripts splicing exons *exs*. We can define a subsumption relationship between segments as *seg*_1_≻*seg*_2_ if and only if *exs*_1_=*exs*_2_,*loc*_1_=*loc*_2_,*Txs*(*seg*_1_)=*Txs*(*seg*_2_) and *w*_1_>*w*_2_. With this relationship we can define the following property of a segment library *S*_*T,L*_

#### **Definition 4**

Maximal Segments For each segment in the set *S*_*T,L*_ to be *Maximal* *seg*_1_≻*seg*_2_⇒*seg*_2_∉*S*_*T,L*_,∀*seg*_1_∈*S*_*T,L*_Thus a maximal segment is the longest common sequence of genomic regions starting at *loc*, such that these regions are spliced similarly, i.e. the entire sequence belongs to the same set of transcripts. That is why in Fig. 1**c** segment S5 is extended to include two exons and its junction, while segment S2 is interrupted by the different splicing of Tx1 and Tx2.

### Segmentation Algorithm

The transcriptome segmentation process can be summarized into three steps: (1) Preprocessing the transcriptome annotation to obtain disjoint exonic bins, (2) Constructing a Segments Graph, and finally (3) Generating the final segments. Transactions in Fig. 1**f** represent these three steps.

1. *Annotation Preprocessing:*

Yanagi applies a preprocessing step to eliminate overlaps present in the transcriptome reference. Parts of an exon (or a retained intron) can be differentially spliced between isoforms either due alternative 3’/5’ splice sites, or transcription start/end sites. For example, splicing the first and second exons between Tx1 and Tx3 in Fig. 1**f**. This step ensures that any splicing event is occurring either at the beginning or the end of a disjoint exonic bin (henceforth, simply ’exonic bin’), which makes the process of generating maximal L-disjoint segments easier. The preprocessing step is independent from the parameter *L*, so it can be done only once per transcriptome reference.

2. *Constructing Segments Graph:*

Currently Yanagi builds a separate segment graph for each gene, since there are no alternative splicing events between transcripts of different genes. However, future work may use segment graphs that connect different genes sharing regions of identical sequence length L or greater, but we have yet to address this.

#### **Definition 5**

Segments Graph A segment graph *G*_*T,L*_ is an acyclic directed graph defined by the pair (*N,E*), where *N* is a set of nodes representing segments, and *E* is the set of directed edges between the nodes. An edge *e*:(*n*_*i*_,*n*_*j*_)∈*E* is created if the segment corresponding to node *n*_*i*_ directly precedes the segment corresponding to node *n*_*j*_ in some transcript.

For each gene, the preprocessed Splice graph is parsed to construct a set of segment nodes (review algorithm details in [38]). These nodes formulate the segments graph of that gene. Each segment node represents an L-disjoint segment, which is not necessarily a maximal segment.

3. *Generating Segments:*

To preserve the maximality property, the segments graph is parsed to aggregated segment nodes into the final maximal segments. In a segment graph, if there is an edge from *node*_*i*_ to *node*_*j*_ while *outdegree*(*node*_*i*_)=*indegree*(*node*_*j*_)=1, that implies that both nodes belong to the same set of transcripts and can be aggregated into a segment that subsumes both nodes. In other words, aggregating nodes along a path in the segment graph bounded by branching points (nodes with indegree or outdegree greater than 1).

Yanagi reports the segments into a FASTA file. Each sequence represents a maximal L-disjoint segment. Each segment sequence has a header specifying metadata of how each segment was formed, including: gene ID, the set of exonic bins *exs* included in the segment, genome location in the first exonic bin of *exs* where the segment starts, genome location in the last exonic bin of *exs* where the segment ends, and the set of transcripts splicing the segment’s region.

### Segment-based calculation of PSI

While Yanagi uses the transcriptome annotation to prepare the segments along with the splicing events, it generates mapping between each event and its corresponding segments spanning the event. For each event, Yanagi takes into consideration the transcripts involved and the event genomic coordinates to decide the set of transcriptome segments that correspond to each of the two possibilities of the splicing event. This step becomes complicated in case of overlapping events. The current version of Yanagi selects segments that spans either the event exon or junctions while the segment belong to at least one transcript that undergoes the corresponding splicing.

After alignment, Yanagi provides segment counts or segment-pair counts in case of paired-end reads. For each splicing event, we calculate the PSI value of event *e* in sample *x* as follows:

1 $$ \begin{aligned} PSI(e, x) = \frac{\tilde{C}_{{inc}}(e,x)}{\tilde{C}_{{inc}}(e,x)+\tilde{C}_{{alt}}(e,x)}; \end{aligned}  $$

2 $$ \begin{aligned} \tilde{C}_{{inc}}(e,x) &= \frac{\sum\nolimits_{s \in S_{{inc}}(e)}SC(s,x)}{\sum\nolimits_{s \in S_{{inc}}(e)}len(s)}, \\ \tilde{C}_{{alt}}(e,x) &= \frac{\sum\nolimits_{s \in S_{{alt}}(e)}SC(s,x)}{\sum\nolimits_{s \in S_{{alt}}(e)}len(s)} \end{aligned}  $$

where *S*_*inc*_(*e*) and *S*_*alt*_(*e*) are inclusion and exclusion segments, respectively, and *SC*(*s,x*) is the segment count in the sample. That means segment-based PSI values uses reads spanning both the junctions and the target inclusion exon towards the inclusion count. In fact, read counts can also include reads extended around the event as far as the segment extends on both sides. This extension takes advantage of situations where multiple splicing events are adjacent, in which the segment approach will include as much discriminative reads into the counts to achieve higher levels of confidence when calculating PSI values.

Finally, as we did here while calculating *PSI* values, one can obtain segment quantification units normalized for sequencing depth and segment length. One way of normalization is to follow similar calculation of *TPM* which is a widely accepted normalized quantification of transcript expressions. However, it may require more sophisticated modeling for length normalization in the presence of complex splicing.

### Simulation Datasets

**Simulation of Switching Abundance (SwitchTx):** We used the simulation data provided by [13] for both fruit fly and human organisms (E-MTAB-3766). Each dataset consists of six samples from two conditions. Each condition has three replicates. The reads for the replicates are simulated from real RNA-seq samples, to get realistic expression values, after incorporating a variance model and the change required between conditions. The simulation is restricted to protein-coding genes in the primary genome assembly. The difference in transcript usage across conditions was simulated in 1000 genes randomly selected from genes with at least two transcripts and high enough expression levels. For each of these 1000 genes, the expression levels of the two most abundant transcripts is switched across conditions. Refer to [13] for full details of the preparation procedure of the dataset.

**Simulation of Incomplete Annotation (IncompTx):** Starting from the transcriptome annotation of the human genome, we searched for candidate cases where one combination of splicing events can be missing from the annotation. For a given gene, a combination of two splicing events (*e*_1_,*e*_2_) can form a candidate case if two conditions are satisfied. 1) If the two splicing events (ordered by their genomic coordinates) have at least one transcript common in their inclusion splicing $T_{1}^{inc} \cap T_{2}^{inc} = T_{c}^{inc}$ while there are no transcripts common between the inclusion of the first event and exclusion of the second event $T_{1}^{inc} \cap T_{2}^{alt} = \phi $ (which will later form the missing isoform in that gene). 2) If the transcript sets $T_{c}^{inc}$ and $T_{2}^{alt}$ share "long enough" contig in the splice graph between the two events. In our simulation, we searched genes on the forward strand for only combinations of SE, A3, A5 typed events. We used a cutoff of 100bp required for the common contig between the two events to be long enough. 2454 genes were found as candidate cases of possible missing isoforms and were used to simulate the data. In each of these genes a single novel isoform is formed by combining the inclusion splicing path of the first event with the alternative splicing path of the second event. Then we used polyester [14] to simulate RNA-seq reads (100bp single end reads) including the novel isoforms which were given high expression levels.

Experiments run throughout the paper used Ensembl GRCh37 and BDGP5 (unless mentioned otherwise) reference genomes and transcriptomes for human and fruit fly annotations, respectively.

## Additional file

Additional file 1 Supplementary Figures and Tables. (PDF 909 kb)

## Data Availability

Yanagi is available at https://github.com/HCBravoLab/yanagi. The repository provides all code scripts required to run the segmentation pipeline to obtain segment counts. z Additionally, We provide ready-to-use segment library (FASTA File) for human (Ensembl GRCh37) and fruit fly (Ensembl BDGP6) transcriptomes at 10.5281/zenodo.2646964. The repository (DOI 10.5281/zenodo.2646964) also provides segment counts and analysis scripts used in the experiments shown in this paper.

## Abbreviations

A3: Alternative 3’splice-site
A5: Alternative 5’splice-site
AS: Alternative splicing
EC: Equivalence class
MX: Mutually exclusive exons
RI: Retained intron
RNA-seq: RNA sequencing
SC: Segment counts
SE: Skipped exon
TCC: Transcript compatibility count
TPM: Transcripts per million
