# Supplementary material for: Yanagi: Fast and interpretable segment-based alternative splicing and gene expression analysis
Source: BMC Bioinformatics. 2019 Aug 13;20:421. doi: 10.1186/s12859-019-2947-6 (PMC6693274; doi:10.1186/s12859-019-2947-6)
Supplement: Supplementary file 1 — Supplementary Figures and Tables. (PDF 909 kb) [file 12859_2019_2947_MOESM1_ESM.pdf]

Supplementary Material for *Yanagi*: Fast and interpretable segment-based alternative splicing and gene expression analysis

Mohamed K Gunady, Stephen M Mount and Héctor Corrada Bravo

June 2019

1 Analysis of Generated Segments

Figure S1 shows the histogram of the lengths of the generated segments compared to the histogram of the transcripts lengths, for each value of L, for both fruit fly (left) and human (right) genomes.

Figure S2 shows how the number of generated segments in a gene is compared to the number of the transcripts in that gene, for each value of L, for both fruit fly (left) and human (right) genomes.

Figure S3 shows the distribution of the coefficient of variation (CV) of the produced segment counts from segments with and without the maximal property. The plot clearly shows that maximal segments have lower CVs to their corresponding short segments (not maximal) for a majority of points (40% of the points has a difference in CVs > 0.05). That corresponds to generating counts with lower means and/or higher variances if the maximal property was not enforced.

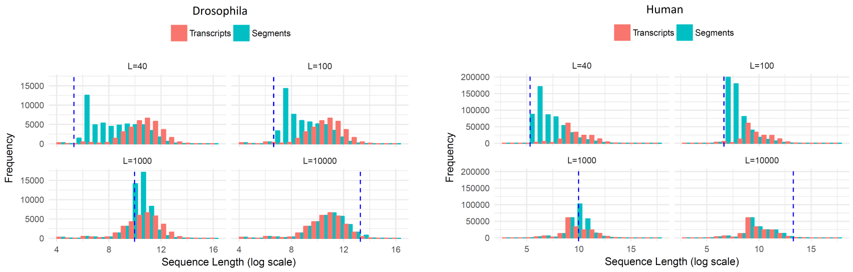

Figure S1: Histogram of transcripts lengths vs. segments lengths for both fruit fly (left) and human (right) genomes, with different values of L (40, 100, 1000, 10,000). Dotted vertical line represents the used value of L during the transcriptome segmentation.

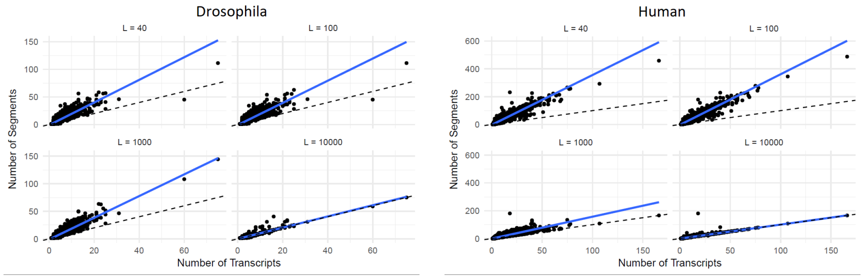

Figure S2: Number of transcripts vs. number of segments, per gene, for both fruit fly (left) and human (right) genomes, with different values of L (40, 100, 1000, 10,000). The figure shows how a fitted line (solid blue) compares to the identity line (dotted black).

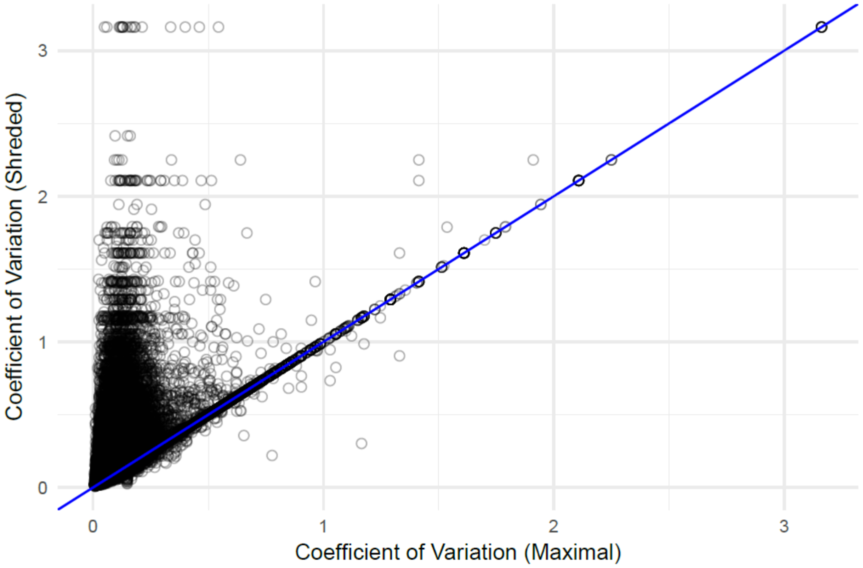

Figure S3: Distribution of coefficient of variation for segment counts produced from maximal segments versus segments without the maximal property enforced. Reads of 10 replicates are simulated from 1000 random genes (with more than two isoforms) in human transcriptome.

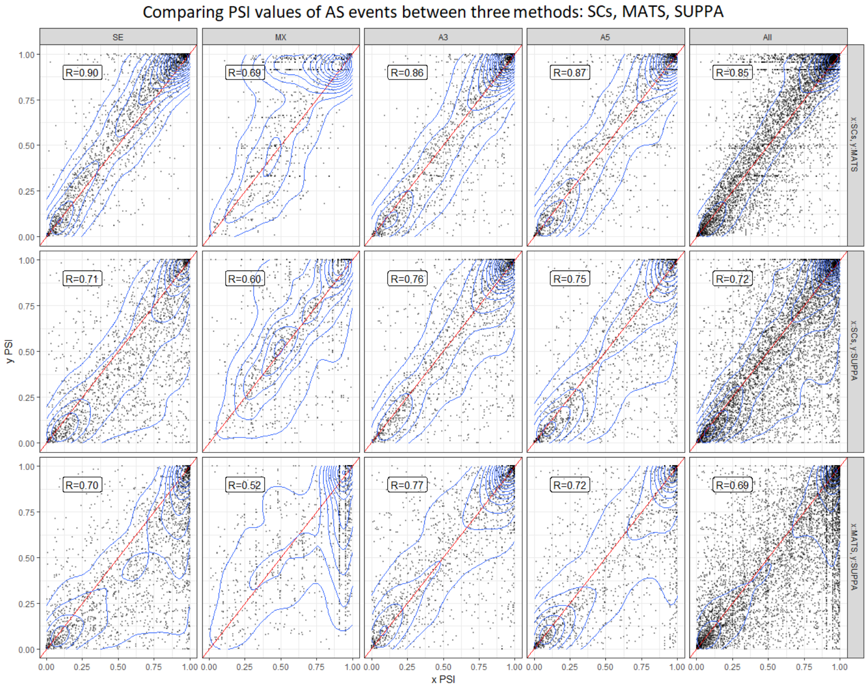

Figure S4: Comparing PSI values calculated using Yanagi (SCs), rMATS and SUPPA. Plots are stratified by event types on drosophila melanogaster sample (SRR3332174).

2 Segment-based Alternative Splicing Analysis

Figure S4 shows a scatter plot of the PSI values of full list of events found in the Drosophila melanogaster transcriptome annotation on RNA-seq dataset of male fly head (available online with GEO accession number GSM2108304) comparing PSI values from three methods: Yanagi, rMATS and SUPPA.

Figure S5 shows scatter plots of the PSI values of filtered events found in human transcriptome annotation on the switchTx dataset comparing PSI values from three methods: Yanagi, rMATS and SUPPA. Figure S6 shows the ROC curves from running differential alternative splicing analysis on the same dataset. Separate plots are shown for non-overlapping events (excludes complex splicings) and events involving transcripts with high expression levels. Table S1 summarizes the number of events subject to the study.

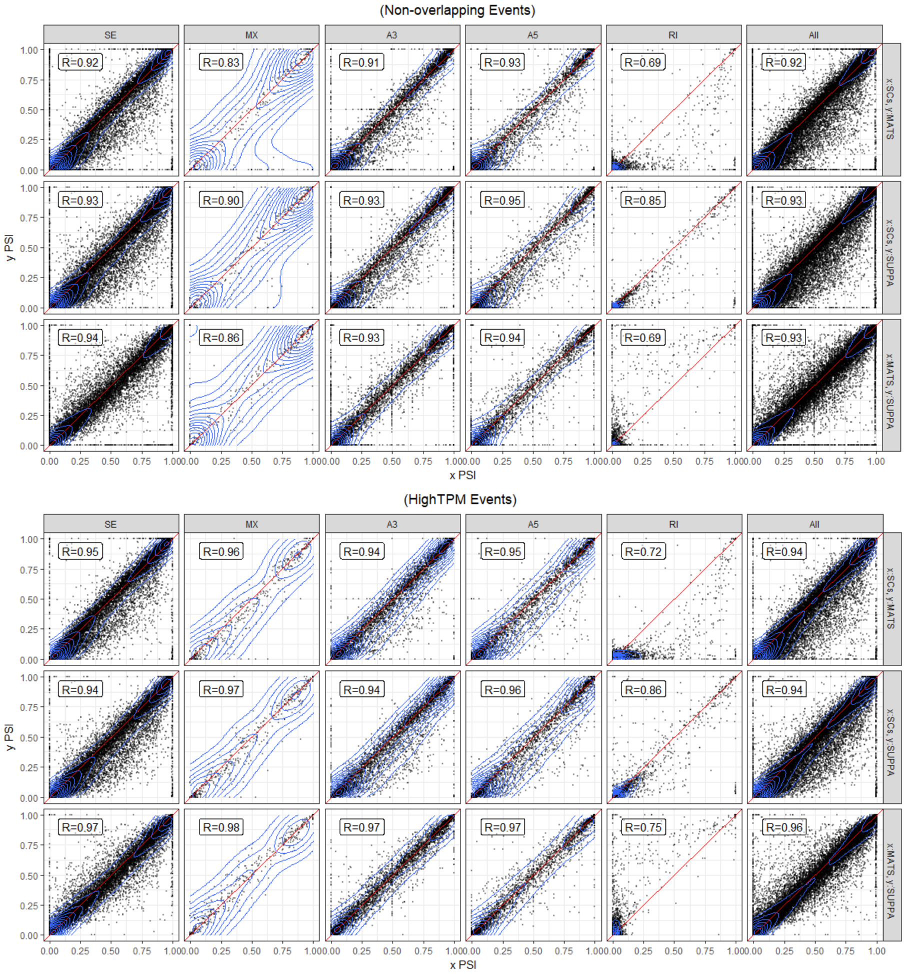

Figure S5: Comparing PSI values calculated using segment counts, rMATS (based on STAR's spliced alignment to genome) and SUPPA (based on estimated TPMs from kallisto's pseudo-alignment and quantification). Plots are stratified by event types. Plots are shown for two subsets of filtered events: non-overlapping events, events with high TPM in the annotation. See Table S1 for number of events of each AS event type shown.

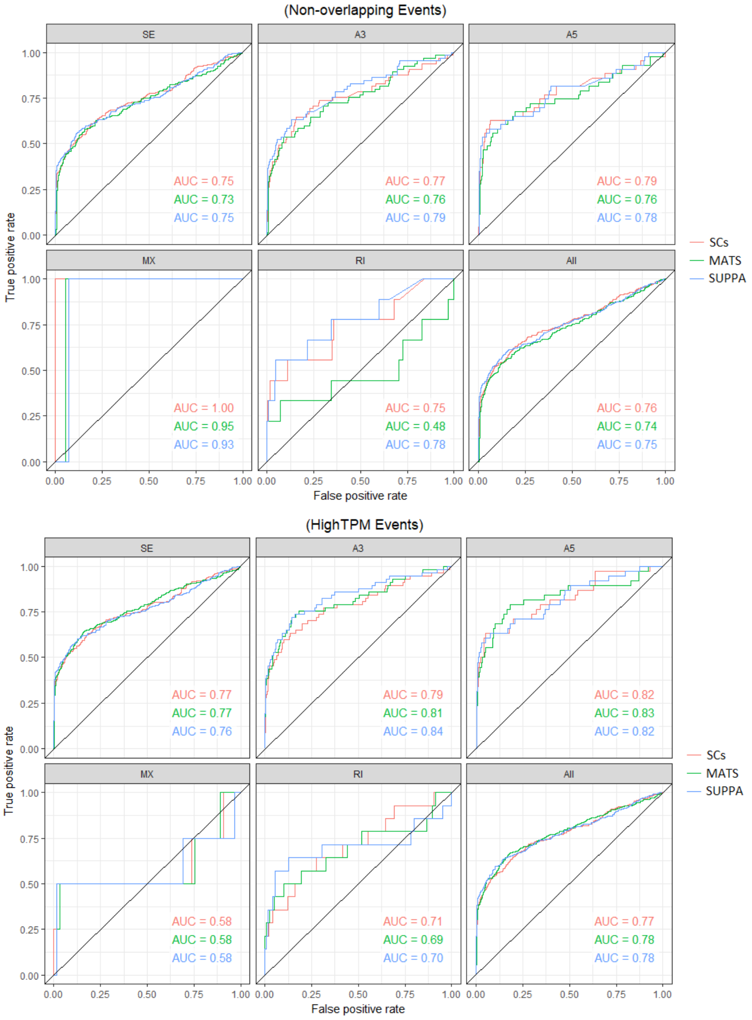

Figure S6: Comparing ROC curves for differential alternative splicing using segment counts, rMATS and SUPPA for simulation dataset of switched abundance. ROC curves are stratified by event types. See Table S1 for number of events of each AS event type shown. Plots are shown for two subsets of filtered events: non-overlapping events, events with high TPM in the annotation.

Table S1: Number of Events in GRCh37 common between MATS and SUPPA for the five event types reported by both tools. Two levels of filtering are applied to obtain three subsets. Non-overlapping events are the simplest events where there is no more splicing other than the two possibilities defining the event. While highTPM events are events where inclusion and exclusion isoform levels are relatively high ($TPM_{inc} > 1, TPM_{ex} > 1$).

Events Subset	SE	MX	A3	A5	RI	Total
Non-overlapping	4,180	68	1,435	885	323	6,891
HighTPM Events	9,756	354	2,327	1,483	793	14,713
All Events	13,650	1,024	3,131	2,053	1,711	21,569

Table S2: Running time per sample (either single or paired-end reads) required by three approaches: using Segment Counts (SCs), counting-based (rMATS), isoform-based (SUPPA). Elapsed time is measured in minutes per pipeline including alignment/mapping step and the generation of PSI values (running using 64 threads on Dual E5-2690 2.90GHz). Both SCs and SUPPA approaches use RapMap for alignment, rMATS uses STAR.

Elapsed Time (mins)	rMATS (STAR)	SCs (RapMap)	SUPPA (RapMap)
Single-End			
Alignment	48	12	12
AS Quant	< 1	< 1	< 1
Paired-End			
Alignment	99	30	12
AS Quant	< 1	< 1	< 1
